# Thermal Shift Assay as a Tool to Evaluate the Release of Breakdown Peptides from Cowpea β-Vignin during Seed Germination

**DOI:** 10.3390/molecules27010277

**Published:** 2022-01-03

**Authors:** Stefano De Benedetti, Camilla Leogrande, Francesco Castagna, Giuditta C. Heinzl, Matias Pasquali, Alessandro L. Heinzl, Daniela Lupi, Alessio Scarafoni

**Affiliations:** Department of Food, Environmental and Nutritional Sciences, University of Milan, 20133 Milan, Italy; stefano.debenedetti@unimi.it (S.D.B.); camilla.leogrande@unigoettingen.de (C.L.); francesco.castagna1@studenti.unimi.it (F.C.); giuditta.heinzl@unimi.it (G.C.H.); matias.pasquali@unimi.it (M.P.); alessandro.heinzl@unimi.it (A.L.H.); daniela.lupi@unimi.it (D.L.)

**Keywords:** seed storage proteins, thermal stability, germination, proteolysis, bioactivity, *Vigna unguiculata*

## Abstract

The present work aimed to characterize the molecular relationships between structure and function of the seed storage protein β-vignin, the vicilin storage protein of cowpea (*Vigna unguiculata*, l. Walp) seeds. The molecular characterization of β-vignin was carried out firstly by assessing its thermal stability, under different conditions of pH and ionic strength, by thermal shift assay (TSA) using SYPRO Orange fluorescent dye. Secondly, its aggregation propensity was evaluated using a combination of chromatographic and electrophoretic techniques. Two forms of β-vignin were considered: the native form purified from mature quiescent seeds, and a stable breakdown intermediate of 27 kDa produced while seeds germinate. TSA is a useful tool for determining and following over time the structural changes that occur to the protein during germination. The main result was the molecular characterization of the 27 kDa intermediate breakdown polypeptide, which, to the best of our knowledge, has never been described before. β-vignin seems to retain its trimeric conformation despite the evident degradation of its polypeptides.

## 1. Introduction

Proteins account for about 22–28% of dry cowpea (*Vigna unguiculata*, l. Walp) seeds [1,2]. Among them, 51% are globulins [3], the main seed storage proteins (SP) of legumes [4,5]. Globulins are usually classified according to their sedimentation coefficients (S) as 7S and 11S proteins, also named vicilins and legumins, respectively. SPs are synthesized during seed development and are deposited inside the cells in specific membrane-bound organelles [4], called protein bodies.

β-vignin is the vicilin protein of cowpea seeds [3]. It is present in two main isoforms with molecular weights of 60 and 56 kDa, differently glycosylated and without disulfide bridges [6].

SPs are traditionally considered the nitrogen reserve that support seedling growth during the first steps of germination [7]. This role has been recently ruled out and new findings indicate that several biological activities become manifest upon proteolytic breakdown. Protein cleavage at germination is believed to occur through a regulated mechanism involving the selective breakdown of specific peptide bonds which, before the native conformation is critically altered to allow the complete hydrolysis, determines the formation of polypeptide fragments [8]. During germination, several preformed and de novo synthesized peptidases act jointly and sequentially [9]. Some of the transiently formed intermediate peptides have been shown to possess specific bioactivities.

Bibliographic data suggest that vicilins have an active role in plant defence processes. For example, a 45 amino-acid-long fragment generated during germination by proteolysis of *Macadamia integrifolia* vicilin has been shown to in vitro inhibit several plant pathogenic fungi [10]. Furthermore, BLAD (*Banda de Lupinus albus Doce*) is a stable intermediary product of vicilin breakdown, which accumulates in cotyledons of *Lupinus* species during germination and shows antibiotic properties [11]. The enzymes involved, and the intermediates generated, are still largely unknown.

Cowpea β-vignin molecular properties, up to now, have been investigated by few authors [3,6,12], and many aspects have not yet been addressed. The full understanding of the structural features of SPs is of primary importance to unveil all their possible biological roles, and to approach applicative uses in various fields, including eco-friendly plant defense, nutrition, and nutraceutics.

Thermal shift assay (TSA) is a tool currently used for rapid, high-throughput identification of stabilizing buffers, additives, and ligands for purified proteins, by monitoring variations of the melting temperature (Tm) of the protein [13]. The Tm assessment is made possible by monitoring the fluorescence emission of a dye, such as SYPRO Orange, which undergoes a significant increase in quantum yield upon binding hydrophobic regions exposed by proteins, undergoing denaturing processes.

We undertook the present study as part of our research activity aimed to characterize the molecular relationships between structure and function of seed storage proteins [14,15,16,17]. The molecular characterization of β-vignin was carried out firstly by assessing its thermal stability, under different conditions of pH and ionic strength, using TSA. Secondly, its aggregation propensity was evaluated using a combination of chromatographic techniques. In order to increase knowledge on the SPs faith during germination, two forms of β-vignin were considered: the native form purified from mature quiescent seeds and the breakdown intermediates produced while seeds germinate.

## 2. Results and Discussion

### 2.1. Solubility

**Figure 1:**
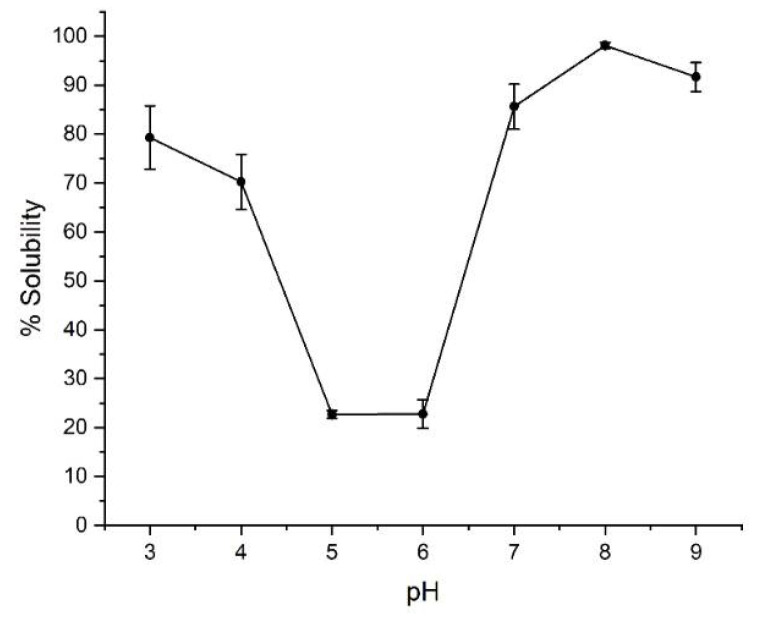
Experimental β-vignin solubility assessment according to pH conditions. See Materials and Methods for buffer composition. The concentration of the protein was 0.5 mg/mL.

As a preliminary characterization, the effect of pH on the protein solubility was tested using different buffer systems with comparable ionic strength (Figure 1). The lowest solubility of the protein was detected between pH 5.0 and 6.0, whereas the highest was above pH 7.0. The predicted isoelectric point of β-vignin is 5.35, as determined by Protparam tool, available on-line at Expasy portal (https://web.expasy.org/protparam/ accessed on 1 December 2021) [18]. The determined pI is in good accordance with that previously reported (pI 5.0) [19].

### 2.2. Thermal Stability of Native β-Vignin and Oligomerization

**Figure 2:**
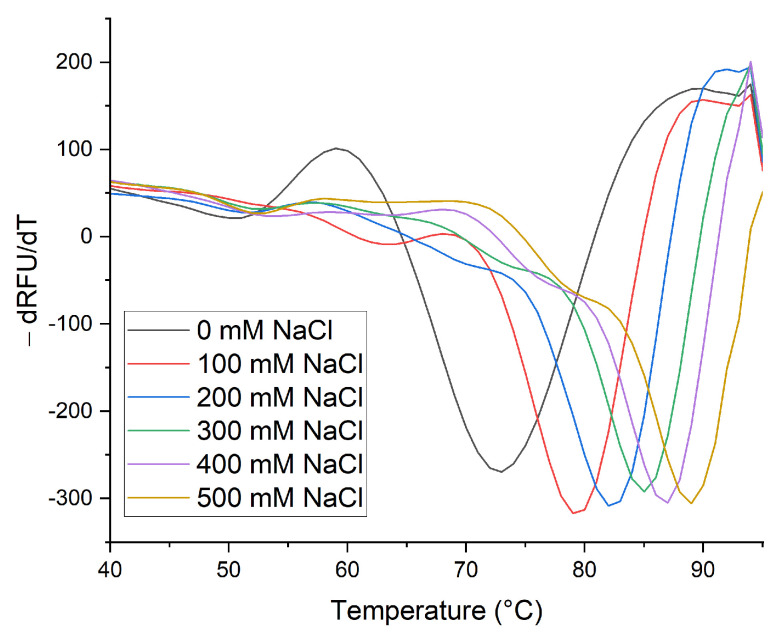
Thermal denaturation of the native protein at pH 7.0 with increasing NaCl concentrations (0 mM; 100 mM; 200 mM; 300 mM; 400 mM; 500 mM). First-order derivatives generated as a function of temperatures.

Thermal stability of native β-vignin was evaluated by thermal shift assay (TSA), testing different pH and ionic strength conditions. TSA was performed using the fluorescent dye SYPRO orange, which undergoes an increase in quantum yield when it binds hydrophobic regions exposed when a protein is thermically denatured. By way of example, the raw denaturation curves and their first-order derivative elaborations of the tests carried out at pH 7.0 are reported in Figure 2. The fluorescence increase midpoint shifts towards higher temperatures as a function of the NaCl concentration, highlighted by the peak obtained with the negative derivative.

The melting temperature (Tm) of the protein was determined from the non-linear fitting to a Boltzmann sigmoidal curve of recorded thermal denaturation data. Points below 50 °C and at post-peak temperatures have not been considered to isolate the sigmoidal transition during denaturation process [13]. The full set of these elaborated data is reported in Figure S1.

**Figure 3:**
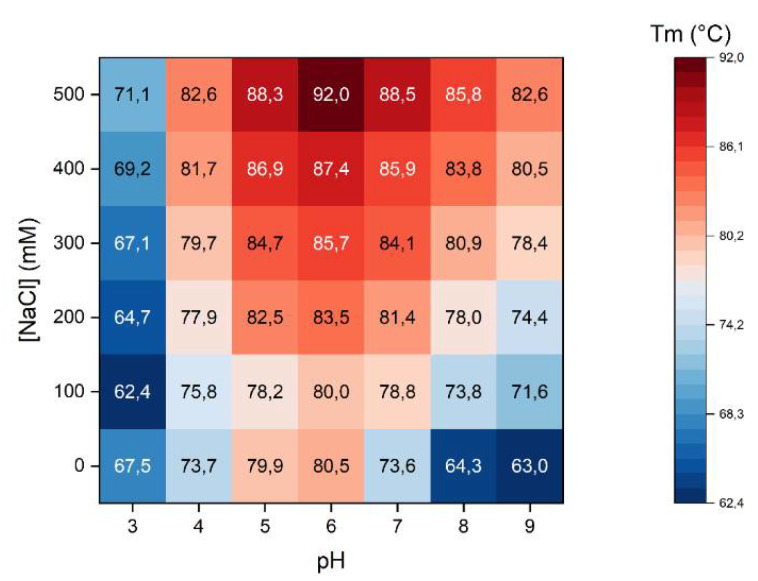
β-vignin melting temperature matrix plot. Each box of the graphical representation reports the calculated melting temperature of the protein at given pH and NaCl concentrations. Values are reported in °C degrees; color code is reported on the right.

Figure 3 reports the calculated Tm values for each tested condition.

As NaCl concentration increased, Tms were higher. This was observed at every pH tested as it could be expected for a protein belonging to globulin class, as β-vignin actually is, whose solubility is enhanced by increasing ionic strengths. Data indicated that the highest stabilities were obtained at pH 5.0 and 6.0. However, the solubility experiment previously shown (Figure 1), evidenced that at these pH levels the solubility of the protein is minimal. The results obtained by TSA should thus be interpreted considering all these data. At a pH around 5.0 and 6.0, β-vignin is likely present as a stable aggregate that undergoes the unfolding transition at temperatures higher than at a pH at which the protein is fully soluble. The formation of these aggregates is likely mediated by hydrophobic interactions which may trap the fluorescent dye even at relatively low temperatures (i.e., room temperature). Raw data curves of these samples (Supplementary Figure S1) indeed show high relative fluorescent unit (RFU) values yet at low temperatures. This indicates that the interaction of the dye with exposed hydrophobic patches occurs in proximity of the protein pI, resulting in a very stable aggregate of native proteins. Considering instead those pH levels at which the protein is soluble, the highest protein stability was obtained at pH 7.0, where even with low NaCl concentrations, Tms values higher than 74 °C were recorded. For samples tested at pH 4.0, 5.0 and 6.0 the standard error was Tm ± 0.3 °C, while at pH 3.0, 7.0, 8.0 and 9.0 the standard error was Tm ± 0.1 °C.

**Figure 4:**
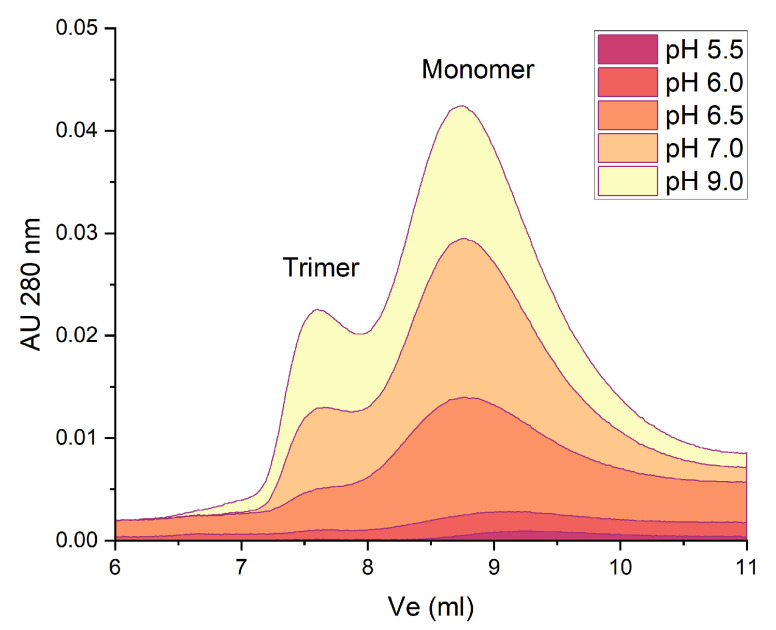
β-vignin trimer–monomer distribution at different pH values determined by SEC using a Superdex S75 10/300 column GL (30 cm × 10 mm). The calculated areas subtended to the peaks are reported in Supplementary Table S1. Protein molecular weight markers used for columns calibration and their elution volumes were the following: ferritin (MW 440 kDa) Ve = V0 = 7.5 mL; bovine serum albumin (MW 67 kDa) Ve = 7.8 mL; ovalbumin (MW 43 kDa) Ve = 10.3 mL; porcine trypsin (MW 23.3 kDa) Ve =13.1 mL; lyosyme (MW 14.2 kDa) Ve = 16.5 mL; porcine insulin (MW 5.7 kDa) Ve = 21.7 mL.

Once we determined the pH and ionic strength conditions at which β-vignin showed maximum stability in solution, we decided to investigate its attitude to oligomerization using a size exclusion chromatography (SEC) approach to shed light on β-vignin monomer to trimer transition as a function of the pH. As a matter of fact, the correlation between pH and oligomerization of seed globulins has been already observed [16,20]. The results are shown in Figure 4. As expected, at a pH of 5.5, close to its isoelectric point, no β-vignin is present in solution. At pH 6.5, where the solubility of the protein rapidly increases (Figure 1), the monomeric form is the predominant species. The trimer/monomer ratio progressively increased at basic pH levels, confirming a pH-dependent oligomerization of β-vignin protein (Table S1).

**Figure 5:**
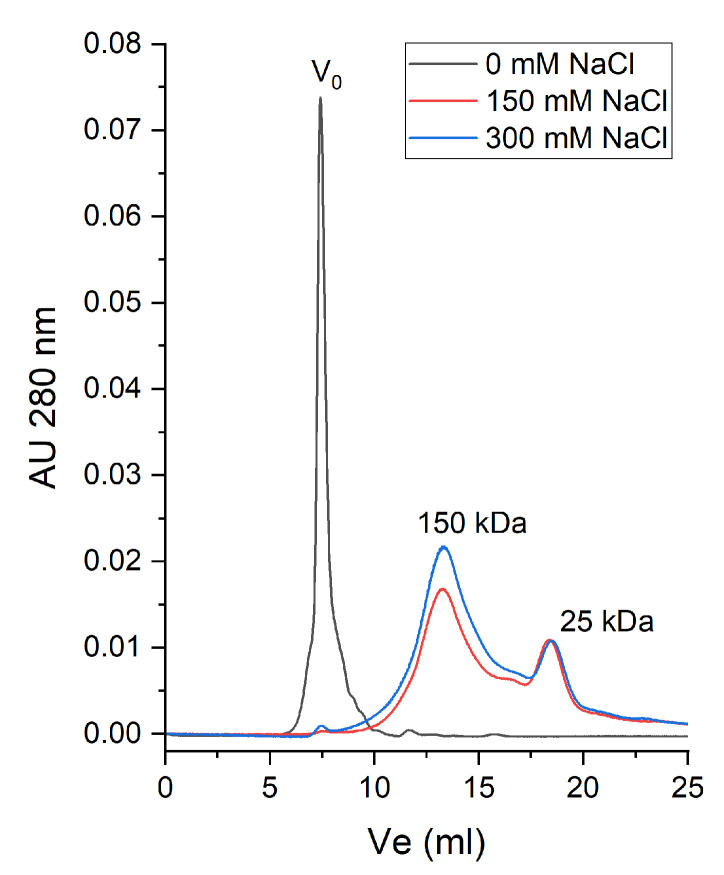
Chromatograms of SEC using a Superdex S200 10/300 GL column (30 cm × 10 mm) of the soluble forms of β-vignin at pH 9.0 containing or not increasing NaCl concentrations. For each run, 50 μg of β-vignin (0.5 mg/mL) was loaded. Calibration of the column with standard proteins was performed in order to determine molecular weights of the eluting peaks. The used proteins and their elution volumes were the following: thyroglobulin (MW 670 kDa) Ve = V0 = 7.6 mL; ferritin (MW 440 kDa) Ve = 10 mL; alcohol dehydrogenase (MW 141 kDa) Ve = 13 mL; bovine serum albumin (MW 67 kDa) Ve = 14.5 mL; trypsin (MW 23.3 kDa) Ve = 17.5 mL.

For further assessing the aggregation propensity of β-vignin, we checked the possible presence of soluble supramolecular complexes at pH 9.0, a value at which the protein is fully soluble. Figure 5 shows that at low ionic strength, almost all the protein eluted with the void volume of the mobile phase (V_0_). Considering the chromatographic matrix (Superdex S200), this should be more than 1000 kDa.

In contrast to what was evidenced by TSA at pH 5.0 and 6.0, this kind of multimerization is likely mediated by electrostatic interactions occurring among β-vignin molecules, rather than hydrophobic interactions. This deduction is based on two considerations. Firstly, TSA did not show any interaction between SYPRO Orange and the protein at temperatures close to room temperature at which the chromatography was performed (Supplementary Figure S1), suggesting that no hydrophobic groups are exposed. Second, increasing the concentration of NaCl to 150 mM led to the complete disassembly of the multimer, leaving one major peak compatible with the size of a β-vignin homotrimer [6]. A higher NaCl concentration (300 mM) did not reveal differences with the lower tested concentration. An extra peak is present in the eluted volume corresponding to a molecular weight of about 25 kDa, probably related to generic contaminant polypeptides or to preformed protein degradation products [4,21]. In order to shed light on these aspects we undertook the following set of experiments.

### 2.3. Proteases from Germinating Cowpea Seeds and β-Vignin Limited Proteolysis

**Figure 6:**
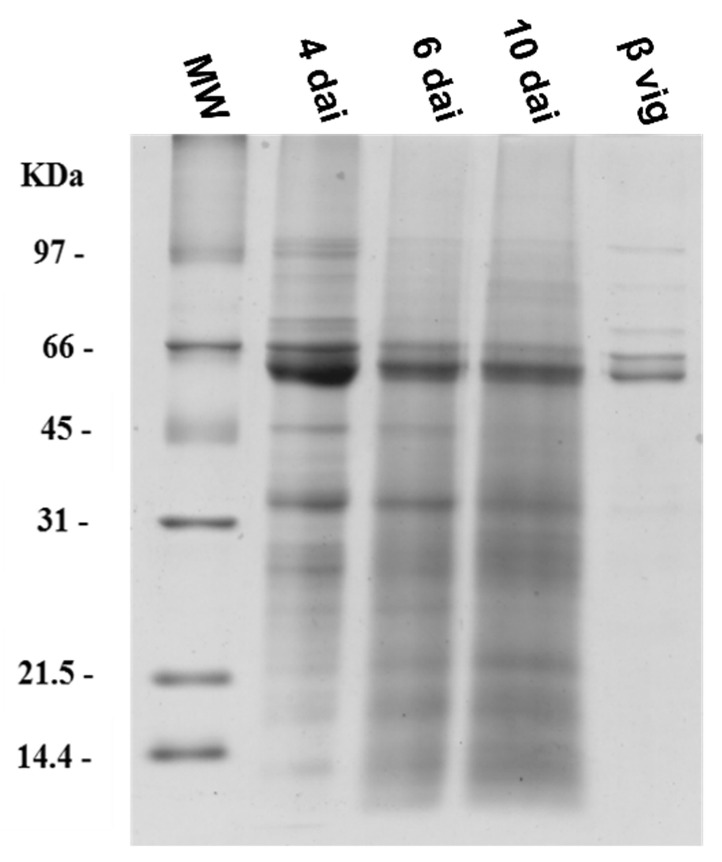
SDS-PAGE of the germinating cowpea extracts obtained after 4, 6, and 10 d.a.i. β-vig: purified β-vignin reference sample. dai.: days after imbibition.

The enzymes involved in such a controlled breakdown process are still unknown and their precise identification was out of the scope of this work. However, we planned to simulate and follow in time course the natural digestion of β-vignin, during the first phases of seed germination, in the attempt to characterize stable intermediate polypeptides. Proteolysis of β-vignin assays were performed using cowpea extracts obtained from germinating seeds at different days after imbibition (d.a.i.). Figure 6 reports the SDS-PAGE separation of the seed extracts obtained after 4, 6, and 10 d.a.i. In all the samples a major band corresponding to β-vignin is evident, mostly at 4 d.a.i., where degradation of the protein is absent or still limited. At 6 and 10 d.a.i. the β-vignin bands are still present but with lower intensity and low-molecular-weight bands appear, indicating the synthesis and presence of proteases acting on storage proteins.

**Figure 7:**
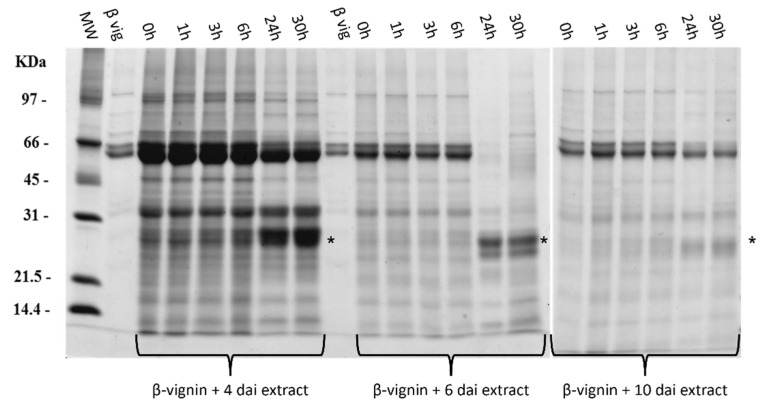
SDS-PAGE of β-vignin digestion with three cowpea germinating seed extracts (4 d.a.i.; 6 d.a.i.; 10 d.a.i.). d.a.i.: days after imbibition. MW: Low-range SDS-PAGE standard. β-vig: purified β-vignin reference sample. Each lane represents a different timepoint of the digestion process. Asterisks (*) indicate the major β-vignin proteolytic product.

In all samples, β-vignin appears as a double band. The two polypeptides differ for their glycosylation status, with the larger one glycosylated and the smaller one non-glycosylated [6]. Purified β-vignin was then incubated with the three described extracts at 37 °C for up to 30 h (Figure 7).

In all the experiments, a time-dependent progressive reduction of the β-vignin band and a corresponding increase of a polypeptide with Mr around 27 kDa occurs. The most pronounced effect was observed with the extract obtained after 6 d.a.i., which left no trace of native β-vignin after 24 h of incubation. On the contrary, proteolysis was less pronounced with the extract obtained at 10 d.a.i. Proteases active on β-vignin are thus transiently expressed, still not markedly present at 4 d.a.i., extensively active at 6 d.a.i., and with a significantly decreased activity at 10 d.a.i. β-vignin incubated without extract was not degraded. The mentioned polypeptide of 27 kDa represents a β-vignin transiently stable core. The presence of other intermediates leading to this fragment was not observed, thus it is likely that proteases slowly degrade small flexible portions exposed to the surface of β-vignin, giving rise to small fragments not retained by the electrophoretic gel, or immediately absolved to their physiological role of nitrogen source for the emerging seedling.

Of note, prolonged incubation times led to the complete disappearance even of the 27 kDa intermediate (Supplementary Figure S2). Thus, the 27 kDa “core” polypeptide may resist exerting potential bioactivities for some time, including seedling defense, and in the end, it is degraded.

### 2.4. Structural Analysis of Proteolysis Main Products

**Figure 8:**
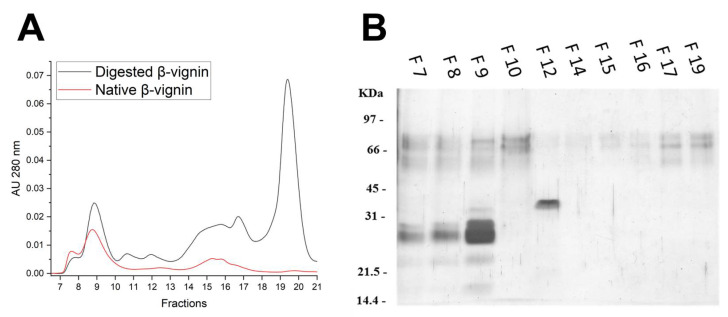
Non-denaturing and denaturing analysis of native and digested β-vignin. Panel (**A**): Superdex S75 10/300 column GL (30 cm × 10 mm) SEC of the native protein (red line) and of the digested protein (black line). For each run, 50 μg of β-vignin (0.5 mg/mL) was loaded. Panel (**B**): SDS-PAGE of the SEC eluted fractions from the digested β-vignin. The gel was silver stained.

SEC was performed to determine under native conditions the molecular size of the β-vignin digestion products with the 6 d.a.i. extract for 30 h. Surprisingly, the chromatograms of not digested and digested β-vignins were perfectly superposable (Figure 8A), indicating a possible similar oligomeric assembly. A shift towards Mrs compatible with the monomeric form was observed (90.2% monomer and 9.8% trimer), whereas the monomer–trimer ratio of native β-vignin at the same pH was 80% and 20%, respectively (Supplementary Table S1). SDS-PAGE analysis of the eluted fractions of digested β-vignin is reported in Figure 8B and confirms the polypeptide composition of the chromatographic peak eluted with a volume similar to that of the native undigested form. Trace amounts of polypeptides corresponding to the intact form of β-vignin are visible in all fractions even, unexpectedly, those eluted with higher volumes.

**Figure 9:**
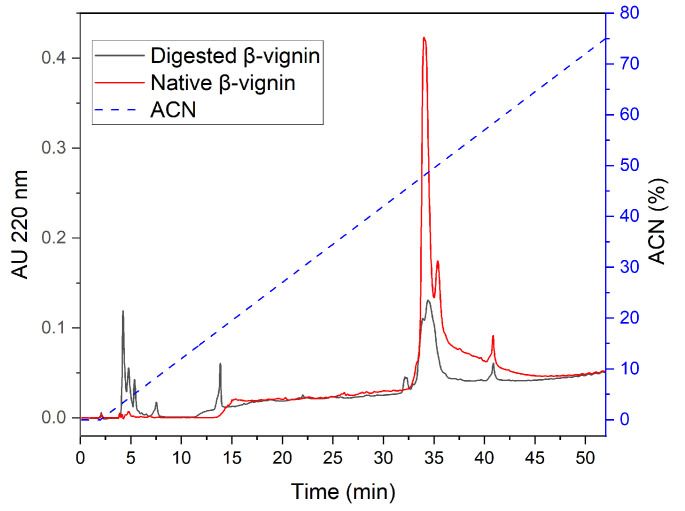
RP-HPLC of the native (red line) and digested (black line) β-vignin. Dashed blue line refers to acetonitrile (ACN) concentration gradient.

To further characterize the products of proteolytic processes, RP-HPLC was performed to compare the native and the 30 h digested β-vignin (Figure 9).

The undigested protein (Figure 9, red line) shows an elution profile where two major peaks are present and elute with an acetonitrile (ACN) concentration, respectively, of 48% and 50%. They likely correspond to the two major β-vignin polypeptides also evident from all the reported SDS-PAGE analyses and previously described [6]. In the 30 h digested sample (Figure 9, black line), additional peaks eluting at lower ACN concentrations appear, as a result of the proteolytic events, and the peaks corresponding to the two major polypeptides decreased. These data corroborate our previous analyses indicating that the two polypeptides do not completely disappear; rather, a proteolysis resistant core remains associated with the proteolytic products generated by the endogenous cowpea proteases.

Digested β-vignin solubility profile (Supplementary Figure S3) was investigated and compared with the previously reported native β-vignin solubility (Figure 1). The lowest solubility was observed at pH 4.0. This means that the proteolytic resistant core is richer in acidic amino acids than the full-length polypeptide, thus the fragments released by the proteases and remaining associated with the 27 kDa core are majorly composed of basic amino acids.

Finally, TSA analyses were performed in order to compare the stability of the complex towards thermal denaturation. If native β-vignin, tested at pH 7.0, shows a Tm of 81.4 ± 0.6 °C, a value that is consistent with the analysis reported above, the digested form at the same pH possesses a Tm of 69.9 ± 0.8 °C, revealing a higher instability due to the proteolytic cleavages. These combined results indicate that the digested β-vignin, despite maintaining an overall structure comparable with the native form, is indeed much more prone to disassembly at the pH tested. Thus, it is likely that the resistant 27 kDa “core” acts as a scaffold to the other proteolytic products that are however available to dissociate and to provide either nutrition to the germinating seed, or to exert some, still unexplored, bioactivity.

### 2.5. Functional Biological Assays

Although the in-depth study of possible biological effects of β-vignin breakdown products was out of the scope of the work, we verified if the residual β-vignin 27 kDa “core” showed different functional properties compared to the native protein, if any. We focused such investigations on two main aspects, namely the possible cytotoxicity on human intestinal undifferentiated Caco-2 cells and the possible modulation of activities relevant to plant defense. The in-depth study of β-vignin functionalities will however the subject of future research.

#### 2.5.1. Effects on Caco2 Cells

Cowpea sprouts can be eaten raw and are becoming popular with some consumers since germination is effective for removing anti-nutritional factors without application of heat processing, which may reduce content of thermal-sensitive nutrients [22]. For this reason, we wondered if the β-vignin could manifest cytotoxicity once ingested. Trypan blue dye is commonly used as a cell stain to assess cell viability and thus for assessing in vitro the cytotoxicity of chemical compounds [23].

The effects were tested on Caco-2 cell line model. The results showed that both intact and digested β-vignin were non-toxic at 0.5 and 1.0 mg/mL concentration. In addition, intact β-vignin was tested also after the pH was lowered to 2.0 for 2 h (in order to denature the protein) and then restored to pH 7.0. Again, no cytotoxic effect was observed (Figure S4).

#### 2.5.2. Effects on Fungal Conidial Germination

The rationale at the basis of this trial is that fragments originated from vicilins resulting from the proteolysis of β-vignin during seed germination could exert a protective activity towards pathogens potentially affecting plant development [14]. The effect towards *F. graminearum* germination of either pure native β-vignin or digested one for 30 h with 6 d.a.i. extract was evaluated. A sample with 6 d.a.i. extract incubated for 30 h alone was tested as a control. Fungal germination was not affected by any of the solutions added to Czapek medium. All conditions tested showed the same germination rate at 8 h, not different from control medium added with peptone (Table S2). No visible conidial modification nor abnormal germination tubules were observed in any of the treatments.

## 3. Materials and Methods

### 3.1. Cowpea Samples

Cowpea seeds (*Vigna unguiculata* (L.) Walp.) were a generous gift from Prof. Ederlan S. Ferreira (College of Pharmacy, Federal University of Bahia, Brazil). β-vignin was extracted and purified as previously reported in Ferreira et al. [6] and lyophilized. Reagents were purchased from Merck Life Science (St. Louis, MO, USA), if not otherwise indicated.

### 3.2. Solubility

β-vignin was dissolved at a concentration of 0.5 mg/mL in buffers at a different pH (citrate buffer, pH 3.0; sodium acetate buffer, pH 4.0; MES-NaOH buffer, pH 5.0 and 6.0; Tris-HCl, pH 7.0, pH 8.0, pH 9.0). The concentration of all buffers was 25 mM. The protein was resuspended by vortexing and underwent four cycles composed of 2 min of sonication and 2 min of rest. The sample was then centrifuged for 10 min at 4°C for 13,000 rpm and the supernatant was used to measure absorbance at 280 nm on a Lambda 2 spectrophotometer (Perkin Elmer, Waltham, MA, USA). Solubility was expressed as percentage ratio with the highest absorbance value.

### 3.3. Chromatography

Salt-dependent aggregation state of β-vignin was evaluated by size exclusion chromatography (SEC) using a Superdex 200 10/300 GL column (30 cm × 10 mm) previously equilibrated with 25 mM Tris-HCl buffer, pH 9.0, with different concentrations of NaCl (0 mM, 150 mM and 300 mM).

pH-dependent oligomerization was evaluated on a Superdex 75 10/300 GL column (30 cm × 10 mm) equilibrated with the previously described buffers at different pH values with the addition of 150 mM NaCl; 25 mM phosphate buffer was used for pH 7.0 analyses.

SEC of the β-vignin digested sample was performed on a Superdex 75 10/300 column GL (30 cm × 10 mm) equilibrated with 25 mM phosphate buffer pH 7.0, 150 mM NaCl. All the analyses were performed with a Waters Delta 600 HPLC system (Waters Corporation, Milford, MA, USA) equipped with a Waters 2487 dual absorbance reader (Waters Corporation, Milford, MA, USA) with a 0.5 mL/min flow rate. Peak integration was calculated with the tool Multiple Peak Fit available in the software Origin (Pro), Version2021b (OriginLab Corporation, Northampton, MA, USA).

Peptide fingerprint after proteolytic digestion was performed with a Reverse Phase Chromatography in a SIMMETRY300 C18 (5 μm) (4.6 × 250) mm column (Waters, Sesto San Giovanni, Italy) fitted on a chromatographic apparatus (Waters) composed of two 510 HPLC Pumps, a 717plus Autosampler and a 996 Photodiode Array Detector. Mobile phase was 0.8 mL/min, mixing solutions A (TFA 0.1% in water) and B (TFA 0.1% in ACN) as follows: 2 min isocratic 100% solution A, 50 min linear gradient to 25% solution A and 75% solution B.

### 3.4. Proteases from Germinating Cowpea Seeds

Cowpea seeds (*Vigna unguiculata* (L.) Walp.) were surface sterilized with 0.5% sodium hypochlorite for 5 min at room temperature under mild shaking. After rinsing 3 times with distilled water to remove leftover traces of sodium hypochlorite, seeds were left overnight at room temperature covered with water. The following day, seeds were placed on wet paper in an aerated box protected from light. At the timepoints of 4, 6, and 10 days after imbibition (d.a.i.), seeds were collected and grounded on an ice-cold mortar and pestle with 25 mM sodium phosphate buffer pH 7.0; 150 mM NaCl was added to the resulting paste in a 1:10 w/v ratio and left shaking at 4 °C for 2 h. The resulting extracts were then centrifuged 13,000 rpm at 4°C for 20 min and frozen at −20 °C. Extracted protein fraction was quantified with the Bradford method [24].

### 3.5. β-Vignin Limited Proteolysis

To evaluate proteolytic activity of the three extracts obtained after 4 d.a.i., 6 d.a.i., and 10 d.a.i., a proteolysis assay was set up. β-vignin at 0.5 mg/mL concentration was prepared in 1 mL 25 mM phosphate buffer pH 7.0, 150 mM NaCl. Then, 0.5 mL of each extract was added and the incubation was performed at 37 °C under shaking. A negative control for the digestion was prepared by adding a β-vignin sample with 0.5 mL of buffer instead of cowpea extracts. For each condition, a sample for SDS-PAGE analysis was collected at the beginning of the incubation (0 h) and at different timepoints (1 h, 3 h, 6 h, 24 h, 30 h).

Digestion of β-vignin prepared in 25 mM phosphate buffer pH 7.0, 150 mM NaCl at 1 mg/mL concentration was performed with a w/w ratio 1:1 with the 6 d.a.i. extract at 37 °C. Samples for SDS-PAGE analyses were collected at 0, 6, 16, 20, 24, 30, 56 and 76 h of incubation. β-vignin without the extract and 6 d.a.i. extract without β-vignin sample were incubated in the same conditions as control samples.

### 3.6. SDS-PAGE

SDS-PAGE was carried out in non-reducing conditions according to Laemmli [25], on 12% polyacrylamide gel. Gels were stained either by Coomassie Blue G-250 (BioRad, Milan, Italy), or silver staining as described by Merril et al. 1981 [26]. Low-range SDS-PAGE Standards (Bio-Rad, Hercules, CA, USA) were used for estimation of Mr.

### 3.7. Thermal Shift Assay (TSA)

Assays were performed with a Bio-Rad CFX Connect Real-Time PCR System (Hercules, CA, USA) into Multiplate 96-Well PCR Plates, low profile, unskirted, clear (Bio-Rad; Hercules, CA, USA). Wells were filled with 30 μL, composed of 5 μL of SYPRO Orange (Sigma-Aldrich; St. Louis, MO, USA) 60×, for a final concentration of 10×, 15 μL of each tested sample at a final protein concentration of 0.5 mg/mL, and the remaining 10 μL with the appropriate buffer (at a 3× concentration). The protocol was set with a temperature analysis range from 20°C to 95°C, with steps of 1 °C increasing every 10 s and fluorescence recorded at the end of each step. All assays were performed in triplicate. Raw fluorescence data were analyzed to determine the melting temperature, from non-linear fitting of thermal denaturation data, as described by Huynh and Partch [13], with the software Origin (Pro), Version2021b (OriginLab Corporation, Northampton, MA, USA). The method was to analyze the stability towards thermal denaturation of the native protein at different pH values (25 mM citrate buffer, pH 3.0; 25 mM sodium acetate buffer, pH 4.0; 25 mM MES-NaOH buffer, pH 5.0 and 6.0; 25 mM Tris-HCl, pH 7.0, pH 8.0, pH 9.0) and NaCl concentrations (0 mM; 100 mM; 200 mM; 300 mM; 400 mM; 500 mM for each pH). The same approach was used to study the stability of proteolysis products at two selected pH values, 3.0 (25 mM citrate buffer, 150 mM NaCl) and pH 7.0 (25 mM phosphate buffer, 150 mM NaCl) after incubation of β-vignin with 6 d.a.i. extract for 30 h.

### 3.8. Effects on Fungal Germination

To assess the effects of the native and digested β-vignin on fungal spore germination, *Fusarium graminearum* strain 3005 [27] was used for fresh conidia production in CMC medium [28]. One thousand conidia in quadruplicate were germinated in a Czapeck medium [29] containing 0.5 mg/mL of pure native protein, or β-vignin digested for 30 h with 6 d.a.i. extract, or 6 d.a.i. extract incubated for 30 h alone. A sample containing 0.5 mg/mL of peptone added to Czapeck medium was set as a control for germination in normal Czapeck medium. Conidia were monitored for 8 h, counting germinating spores at 2, 4, 6, and 8 h under a microscope. Results are reported as % of germinated.

### 3.9. Caco-2 Cells Viability

The impact of cowpea proteins on cell viability was measured with Trypan blue assay [23]. Briefly, cells were seeded in 24-well plate at a density of 5 × 10^4^ cells/well in 500 µL of culture medium. At least three replicate wells were conducted per sample. After 48 h of incubation, proteins were added to all wells and the cells were incubated for further 48 h. Next, the media were removed, cells were washed with 200 µL of PBS and 100 µL of trypsin was added to each well. After 5 min, 100 µL of DMEM was added to stop the reaction, all media was aspirated and centrifuged to remove any trypsin. Cell suspension was collected and 500 µL of DMEM was added. A 1:1 dilution of cell suspension and Trypan blue dye was prepared, 10 µL of Trypan blue/cell mixture was added to a counting slide and counted with a TC20 automated cell counter (Bio-Rad, Hercules, CA, USA). Cell viability of treated cells was calculated by comparing viability of treated cells with viability of control cells (100% viable cells). Cell viability was calculated as following: (Sample cell viability)/(Control viability) × 100.

### 3.10. Statistical Analysis

When appropriate, all determinations were carried out in triplicate, unless otherwise specified. Data are expressed as means ± S.D. Data were analyzed by *t*-tests. values < 0.01 were considered statistically significant.

## 4. Conclusions

The present work investigated the stability and aggregative properties of both native β-vignin and proteolytic products of β-vignin transiently accumulated during seed germination, at different pH and ionic strength conditions. TSA has proved to be a useful tool for determining and following over time the structural changes that occur during germination. The main result was the molecular characterization of the 27 kDa intermediate breakdown polypeptide, which, to the best of our knowledge, has never been described before. β-vignin seems to retain its trimeric conformation despite the evident degradation of its polypeptides. The molecular characterization of β-vignin is useful to approach the investigation of its functional role during germination. In fact, its proteolytic degradation may lead to generation of proteolytic intermediates with different properties, such as insecticidal or antifungal actions. Other functions, however, cannot be ruled out. For example, very preliminary results seem to indicate that the two tested β-vignins may act differently on the fitness of *Drosophila melanogaster*. Although the results on the possible modification of any functional properties are far from being exhaustive, our results seem to indicate that the digestion of the protein that occurs during the early stages of germination does not lead to the formation of toxic degradation intermediates, at least in the adopted experimental conditions. This will certainly be the subject of further studies and investigations, including tests on model entomological species.

## Data Availability

Data are contained within this article.

## Supplementary Materials

The following are available online, Figure S1: β-vignin melting temperature raw data matrix plot; Figure S2: SDS-PAGE of β-vignin digested with 6 d.a.i. extracts for prolonged incubation times; Figure S3: Experimentally determined solubility of β-vignin digested for 30 h with 6 d.a.i. extract; Figure S4: Cytotoxicity of native and digested β-vignin on Caco-2 cells; Table S1: Monomer–trimer distribution at different pH levels of native β-vignin; Table S2: *F. graminearum* conidia germination at 8 h after seeding.
