# Supplementary material for: Thermal Shift Assay as a Tool to Evaluate the Release of Breakdown Peptides from Cowpea β-Vignin during Seed Germination
Source: Molecules. 2022 Jan 3;27(1):277. doi: 10.3390/molecules27010277 (PMC8746429; doi:10.3390/molecules27010277)
Supplement: Supplementary file 1 [file molecules-27-00277-s001.zip › molecules-1516831-supplementary.pdf]

molecules

MDPI

Supporting Information

Thermal Shift Assay as a Tool to Evaluate the Release of Breakdown Peptides from Cowpea β-Vignin during Seed Germination

Stefano De Benedetti, Camilla Leogrande, Francesco Castagna, Giuditta C. Heinzl, Matias Pasquali, Alessandro L. Heinzl, Daniela Lupi and Alessio Scarafoni

Table S1. Monomer – trimer distribution at different pHs of native β-vignin. The ratio of each form was calculated as the % area under each peak as depicted in the Figure 4 of the manuscript. See Methods (paragraph 3.3) for details.

pH	Monomer (%)	Trimer (%)
9.0	75	25
7.0	80	20
6.5	91	9
6.0	94	6
5.5	insoluble	

Table S2. *F. graminearum* conidia germination at 8 hours after seeding. Results are reported as % of germinated conidia. Experiments were performed in quadruplicate Tested samples were included in the medium at 0.5 mg/mL concentration each.

Sample name	Conidia germination (%)	SD (%)
Native β vignin	89.5	1.7
Digested β vignin	89.0	1.4
Extract 30 hours	90.8	0.9
Control peptone	90.5	1.7

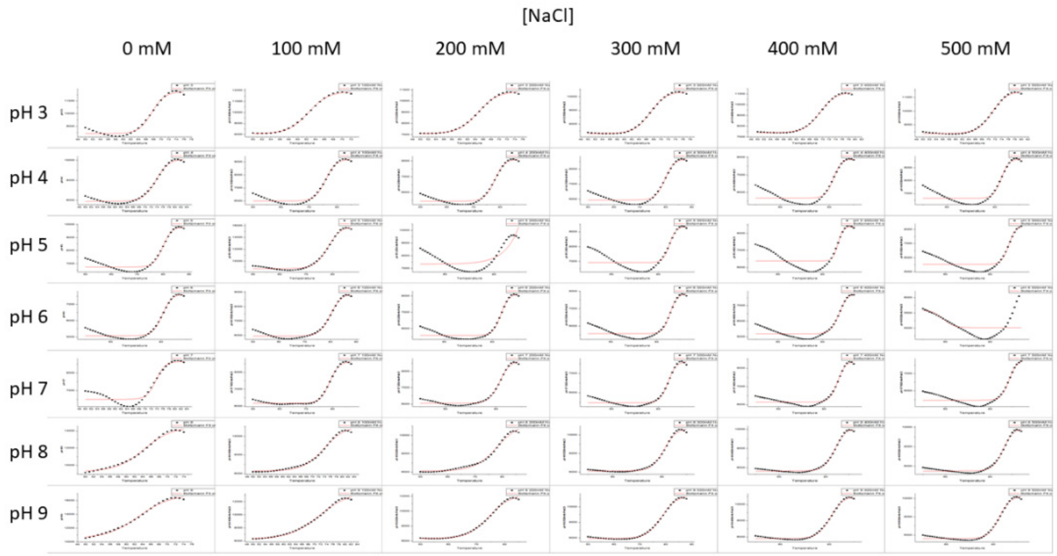

Figure S1. β-vignin melting temperature data matrix plot. Each curve is built in order to highlight the transition point of the non linear fitted data corresponding to the melting temperature of the protein in each condition. Data have been truncated below 50°C and at post-peak temperatures in order to isolate sigmoidal transition during denaturation process. The x axis represents temperature data and y axis reports Relative Fluorescence Units (RFU).

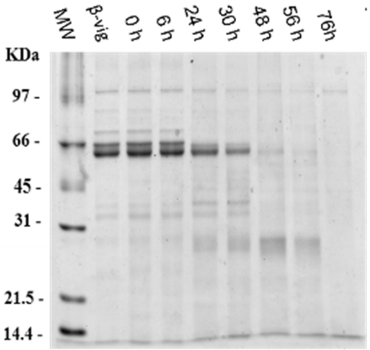

Figure S2. SDS-PAGE of β-vignin digested with 6 d.a.i. extracts for prolonged incubation times. MW: Low-range SDS-PAGE Standard. β-vig: purified β-vignin reference sample. Each lane represents a different timepoint of the digestion process.

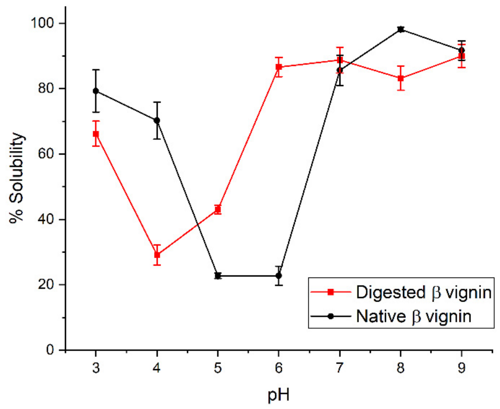

Figure S3. Experimentally determined solubility of β-vignin digested for 30 hours with 6 d.a.i. extract (red line). The solubility of native β-vignin (black line) is also reported for comparison. Either the pI and the minimum solubility of the digested β-vignin shifts towards more acidic pHs than the corresponding ones of the native protein.

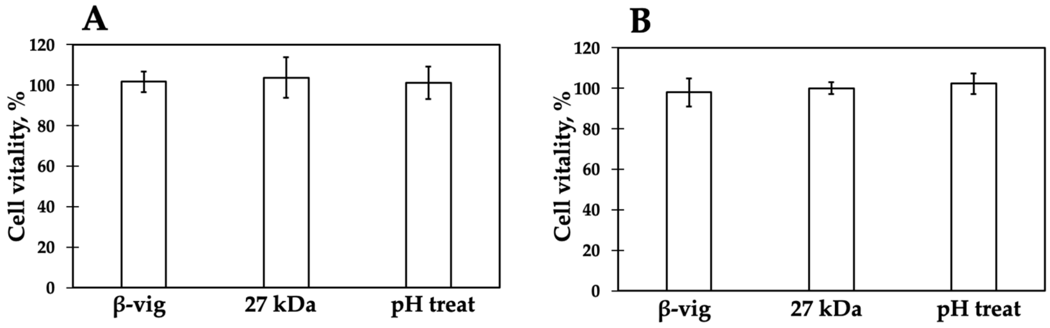

Figure S4. Cytotoxicity of native and digested β-vignin on Caco-2 cells. Panel A: tested proteins were 0.5 mg/mL. Panel B: tested proteins were 1.0 mg/mL. and digested tested. β-vig: native β-vignin. 27 kDa: β-vignin digested for 30 hours with 6 d.a.i. extract. pH treat: intact β-vignin tested after the pH of the solution was lowered to 2.0 for 2 hours and then re-stored to pH 7.0.
